# Molecular Characterization of Antibiotic Resistance Associated with *TEM* and *CTX-M* *ESBL* in Uropathogenic *E. coli* Strains Isolated from Outpatients

**DOI:** 10.30699/IJP.20201.521669.2556

**Published:** 2021-07-06

**Authors:** Sorour Farzi, Reza Ranjbar, Mohammad Niakan, Mohammad Hossein Ahmadi

**Affiliations:** 1 *Department of Microbiology, Faculty of Medicine, Shahed University, Tehran, Iran.*; 2 *Molecular Biology Research Center, Baqiyatallah University of Medical Sciences, Tehran, Iran*

**Keywords:** E. coli, urinary tract infection, ESBL, antibiotic resistance

## Abstract

**Background & Objective:**

*Escherichia coli *(*E. coli*) is a leading cause of urinary tract infections becoming resistant against beta-lactams and cephalosporins through different mechanisms, including *ESBL* production due to the presence of *ESBL* specific genes, including *blaCTX-M* and *blaTEM*. The purpose of the present study was to detect the uropathogenic *E. coli *strains producing the *ESBL*.

**Methods:**

A total of 100 isolates of uropathogenic *E. coli* were randomly selected in a period of 6 months and their resistances to a number of antibiotics including amoxicillin, amikacin, gentamicin, ciprofloxacin, ceftazidime, cefotaxime, ceftriaxone, ceftizoxime, nalidixic acid, and nitrofurantoin were determined. Then, DDT test was used to detect the presence of *ESBL*. Finally, the presence of *blaCTX-M* and *blaTEM* resistance genes was analyzed by PCR method.

**Results:**

The resistance profile of bacterial isolates to the antibiotics was as follows: amoxicillin: 16.7%, amikacin: 7.8%, gentamicin: 20.3%, ciprofloxacin: 35.5/%, ceftazidime: 35.0%, cefotaxime: 40.0%, ceftriaxone: 41.3%, nalidixic acid: 64.0%, nitrofurantoin: 9.7%, and ceftizoxime: 100%. Of these, 28 isolates (28%) were reported to be resistant to cefotaxime, ceftazidime, and ceftriaxone. In DDT test, 21 *ESBL *positive cases (21%) were detected. PCR results showed that the presence of *blaCTX-M* and *blaTEM* genes in the isolates were 21% and 20%, respectively.

**Conclusion:**

Regarding the production of *ESBL* by some *E. coli* isolates, phenotypic detection of *ESBL-*producing isolates is routinely suggested in the laboratories. Likewise, the treatment regimen should be selected regarding the *ESBL* production to avoid treatment failure.

## Introduction

Beta-lactam antibiotics are the common drugs in the treatment of bacterial infections (1). These antibiotics have been widely used since 1980 in order to treat serious infections caused by gram-negative bacteria but resistance against this group of antibiotics happened quickly around the world (2-4). Production of beta-lactamase enzymes is the main mechanism by which the resistance to these antibiotics occurs (5). These enzymes hydrolyze the core of beta-lactam antibiotics and finally inactivate them. The emergence of new antibiotics such as broad spectrum cephalosporins, aztreonams, and their wide usage in the treatment of bacterial infectious diseases has led to the presence of a new class of enzymes called extended-spectrum beta-lactamases (*ESBL)* (6, 7). *ESBL* enzymes are often coded by large plasmids that contain resistance genes to several antibiotics such as aminoglycosides, trimethoprim, sulfonamides, tetracycline, and chloramphenicol. The resistance to several antibiotics is an important feature of the* Enterobacteriaceae* strains producing *ESBL* (8).

*ESBLs *are classified into four main classes from A to D (Ambler classification). *ESBL* class A includes beta- lactamases such as *CTX-M*, *TEM*, *SHV,* and their subtypes. *CTX-M* enzymes constitute a specified category of beta-lactamases class A that is growing at a rapid rate. The first *CTX-M* enzyme that was isolated from clinical isolates was *CTX-M-1* isolated from Enterobacterial strains in Europe in the late 1980s (9). After that, a lot of new variants of *CTX-M* were defined, so that more than 50 variants were described based on differences in the amino acid sequence that were divided into 5 groups or sub-types including *CTX-M-1*, *CTX-M-2*, *CTX-M-8*, *CTX-M-9*, and *CTX-M-25* (10, 11). *CTX-M* beta-lactamases are part of natural *ESBLs* which hydrolyze cefotaxime or ceftriaxone more than ceftazidime. Therefore, most of the* CTX-M*-producing strains show significant resistance against ceftazidime and cefotaxime (12, 13).

The *TEM-1* enzyme was originally found in *E. coli* isolated from blood culture of a Greece patient called Temoniera and hence was named *TEM *(13). This enzyme caused resistance to penicillin and first genera-tion cephalosporins such as cephalothin and cephalo-ridine (14). Various types of *TEM* beta-lactamases have been obtained through substituting amino acids in the activation site of *TEM* so that more than 130 types of *TEM* have been identified (15). The prevalence of some of these enzymes is different in various parts of the world (16). Today, the number of organisms producing *TEM *enzymes has been increased, while this has been considered as a crisis in the treatment of infections caused by these bacteria (17).

Since the prevalence of drug resistance is increasing and it has delayed the treatment of patients, especially those admitted to hospitals, the present research was performed to assess the detection and treatment of infections caused by *E. coli* strains being resistant to beta-lactam antibiotics in Tehran's Baghiyatallah Hospital.

## Material and Methods

Isolation of Bacteria from Urine Specimens

This was a cross-sectional descriptive study. In a period of 6 months, 100 urine samples were collected from patients referred to Baghiyatallah Hospital of Tehran, and cultured on selective media of Eosin Methylene Blue (EMB) agar. Then, the plates were incubated at 37°C for 24 hours. The colonies grown underwent biochemical tests using MRVP broth, Simon citrate agar, TSI agar, SIM agar, MacConkey agar as well as Urea broth media. Finally, the *E. coli* isolates were identified and the corresponding colonies were kept at -70°C in the Skim Milk medium to be used in the later stages.

Identification of ESBL-producing *E. coli* Strains 

The pattern of antibiotic susceptibility was studied by disk diffusion (Kriby-Bauer method) according to the instructions of Clinical and Laboratory Standards Institute (CLSI) using antibiotic disks manufactured by MAST Company (UK) including cefotaxime (30 mg), ceftriaxone (30 mg), ceftazidime (30 mg), cefpod-oxime (30 mg), nalidixic acid (10 mg), ceftizoxime (30 mg), cefixime (30 mg), nitrofurantoin (10 mg), ami-kacin (10 mg), and gentamicin (10 mg) (18). In order to perform disk diffusion, at first microbial suspensions were prepared as 0.5 McFarland from 18-hour colonies and then cultured on the surface of Mueller-Hinton agar (Merck, Germany), using a sterile swab. After 15 minutes, antibiotic disks were placed on the surface of the media every 20 mm from each other. After 15 min-utes, plates were incubated at 35 to 37°C for 16 to 18 hours. The diameter of inhibition zone was measured and interpreted according to the criteria of CLSI. Iso-lates with inhibition zone diameters of ≤22, ≤25, and ≤27 for ceftazidime, ceftriaxone, and cefotaxime, respectively, were evaluated for the presence of *ESBL* (18).

Phenotypic Confirmatory Test

To confirm *ESBL* production in candidate organisms, phenotypic confirmatory Double Disk Test (DDT) was used according to the CLSI command. The hybrid disks containing ceftazidime (30 mg) + clavulanic acid (10 mg) and cefotaxime (30 mg) + clavulanic acid (10 mg) were prepared from MAST Company. Afterward, the plates were incubated at 37°C for 24 hours. Then the diameter of inhibition zone was measured using a millimeter ruler and the results were interpreted according to the CLSI standards.

If the inhibition zone diameter of a colony around the combination disk was at least 5 mm bigger than the inhibition zone diameter of the single disk of the same antibiotic, it was considered as *ESBL*-producing isolate (18). In this test, the bacteria *E. coli* with the code of ATCC25922 and *Klebsialla pneumonia* with the code of ATCC700603 were used as *ESBL* positive and negative controls, respectively.

DNA Extraction and PCR

After performing the initial phenotypic con-firmatory test, the samples containing resistant isolates were selected for DNA extraction. Boiling method was used for DNA extraction (19) and PCR reaction was done to detect beta-lactamase genes including *bla-CTX-M* and *bla-TEM* under the conditions presented in Table 1. The forward and revers primers used in the present study were designed using the Primer3 software and their specificity was determined by Primer-blast online software (http://www.ncbi.gov/-tools/primer-blast). Moreover, to assure the quality of the primers, the Oligo analyzer program was used. The reaction mixture in a volume of 20 ml contained: 10 ml of master mix, 2 microliters of forward and revers primers (20, 21) (Table 2), 1 mL of extracted genome, and 7 mL of sterile distilled water.

Finally, electrophoresis of PCR products was performed in order to identify specific fragments with the sizes of 593bp (for *blaCTX-M* gene) and 867bp (for *bla-TEM* gene) on a 1.5% agarose gel with a size marker of 100 bp.

## Results

The percentage of the identified isolates being resistant to the antibiotics are listed in Table 3.

Based on the results of disk diffusion screening test, 28 samples (28%) were resistant to cefotaxime, ceftazidime, and ceftriaxone, simultaneously. In DDT test, 21 cases were confirmed to be *ESBL* producing isolates.

**Fig. 1:**
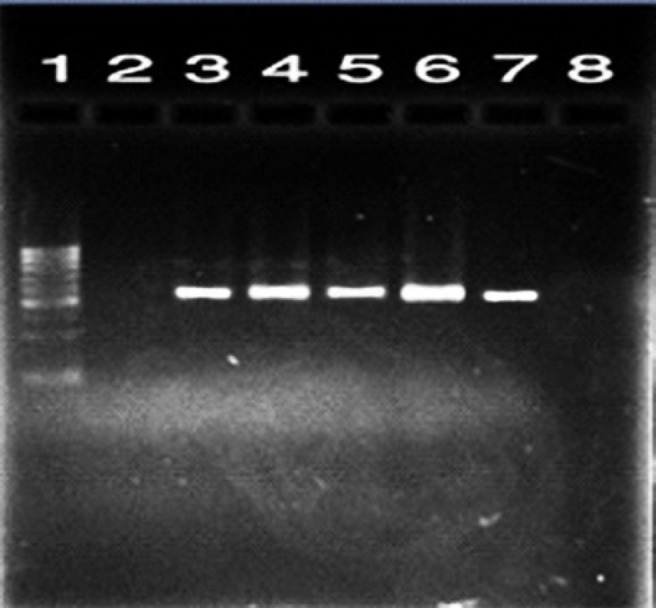
Electrophoresis of PCR products for *blaCTX-M* gene. Lane 1: size marker (100 bp DNA ladder); lane 2: negative control, lane 3: positive control for *blaCTX-M* gene; lanes 4-7: PCR positive samples (593 bp) for *blaCTX-M* gene; lane 8: negative sample with no band

**Fig. 2:**
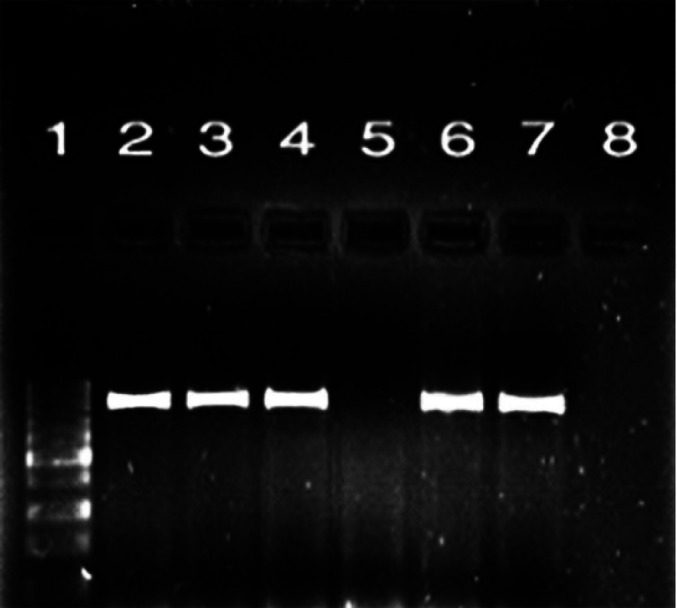
Electrophoresis of PCR products for *blaTEM* gene. Lane 1: size marker (100 bp DNA ladder); lane 2: positive control for *TEM* gene; lanes 3, 4, 6, and 7: PCR positive samples for *bla-TEM* gene (867bp); lane 5: negative sample with no band; lane 8: negative control

According to the PCR results, the presence of *blaCTX-M* and *blaTEM* genes in 21 *ESBL* positive isolates were 21% and 20% for mentioned genes, respectively (Figures 1 and 2). Additionally, 19 samples (19%) contained isolates harboring both of the genes.

**Table 1:** PCR condition for amplification of ESBL gens

Cycle repeats	Time (min)	Temperature (˚C)		Step	
		*blaCTX-M*	*blaTEM*		
1	5	96	95	**1. Initial denaturation**	
30	1	96	94	Denaturation	**2. Amplification**
	1	60	52.2	Annealing	
	1	72	72	Extension	
1	7	72	72	**3. Final Extension**	

**Table 2:** **The **primers used for PCR

Primer	5´-sequence-3´	Expected-size (bp)	Reference
TEM-F TEM-R	ATGAGTATTCAACATTTCCG CTGACAGTTACCAATGCTTA	867	
CTX-M-F CTX-M-R	ATGTGCAGYACCAGTAARGT TGGGTRAARTARGTSACCAGA	593	

**Table 3:** Antibiotic resistance pattern for the examined antibiotics

Antibiotic	Number of resistant isolates (%)
Amoxicillin	2 (16.7)
Amikacin	4 (7.8)
Gentamicin	12 (20.3)
Cephalexin	5 (29.4)
Ceftazidime	21 (35.0)
Ceftriaxone	26 (41.3)
Cefotaxime	4 (40.0)
Ceftizoxime	2 (100)
Nitrofurantoin	6 (9.7)
Nalidixic acid	32 (64.0)
Ciprofloxacin	22 (35.5)

## Discussion

Bacterial beta-lactamase genes, especially *ESBLs* genes are among the effective factors that increase their resistance to beta-lactam antibiotics such as broad-spectrum cephalosporins. *ESBL* producing bacteria have created many health problems in recent years and novel methods are required to detect these bacteria in the clinical microbiology laboratories (21, 22). *ESBL* phenotypic detection is an appropriate method for differentiation between *ESBL*-producing isolates and isolates that use other mechanisms of beta-lactam antibiotics resistance (23). In the present study, among the analyzed samples, 21 samples (21%) were *ESBL* producers which indicate a high rate of *ESBL*-producing *E. coli *isolates in patients with urinary tract infection. These isolates were susceptible to the clavulanic acid (a broad spectrum beta-lactamase) and one case was not susceptible to this antibiotic which could be due to the production of enzymes such as AmpC (24). The prevalence of *ESBL *production in *E. coli-* and *Klebsiella pneumonia-*positive samples is different in various countries; for example, in Korea, the prevalence of these organisms is in the range of 4.8%-7.5% and 22.5%-22.8% for *E. coli* and *Klebsiella pneumoniae,* respectively. In India, the frequency of *ESBL* production is 34.2% and 27.3% for *E.coli* and *Klebsiella pneumoniae*, respectively (25). These results had no statistically significant difference compared to our results.

Several studies have indicated that *ESBL* production by nosocomial and non-nosocomial *E. coli* strains, has been quickly spread around the world, due to the emergence of *ESBL* type *CTX_M*. Lewis *et al*., in 2007 using phenotypic tests and molecular techniques, examined *ESBL* production on 94 urine samples of patients infected with uropathogenic *E. coli* and reported that among the samples, the most common *ESBL* type was *CTX-M *(26).

In the present research, in line with the other studies, susceptibility to gentamicin in the isolates producing *CTX-M* was more than that in the isolates producing *TEM*. Recent studies in Canada, Italy, Spain, Greece, and the United Kingdom revealed that *ESBL* production in *E*. *coli*, especially those that produce *CTX-M* have multiple resistances to trimethoprim, Sulfamethoxazole, tetracycline, gentamicin and ciprofloxacin (27).

Pitout *et al.* (2005) found that *Enterobacteriaceae* (like *E. coli*) isolated from urinary tract infections that produce *ESBL* type *CTX-M* are resistant to quinolones; in other words, resistance to quinolones is often associated with *CTX-M* (27). This relationship is confirmed in our study as in the isolates having *CTX-M*, 11 cases with resistance to ciprofloxacin were observed while was not observed in any other type of *ESBLs*. Moreover, the isolates in our study had the characteristics of *ESBL*-mediated resistance because a high percentage of isolates were resistant to the third generation cephalosporins, including: 35%, 41.3%, 40%, 100%, 50%, and 66.7% for ceftazidime, ceftriaxone, cefotaxime, ceftizoxime, cefixime and cefpodoxime, respectively. In a number of countries such as Iran, cephalosporins are the antibiotics of choice using to treat UTIs and practitioners use these antibiotics in abundance. 

In a study conducted by Pour Akbari *et al.* in 2012, 100 *E. coli* strains isolated from UTI in patients aged 2 to 12 years underwent antibiotic susceptibility testing using disk diffusion method. Their results indicated a high percentage of susceptibility of *E. coli* to antibiotics including amikacin and nitrofurantoin (95% and 91%, respectively) (28). Meanwhile, in another research in the United States, the sensitivity to antibiotics obtained as 90% (29). We found similar results in our study regarding the susceptibility to the two antibiotics, amikacin (72.6%), and nitrofurantoin (83.9%). 

## Conclusion

The use of amikacin as empiric therapy is a good choice for patients with urinary tract infection as well as the use of nitrofurantoin as an antibiotic to prevent urinary tract infections. Moreover, concerning the production of *ESBL* by some isolates, phenotypic detection of *ESBL*-producing isolates is routinely suggested. The treatment should be selected regarding the *ESBL* production to avoid treatment failure.
